# Ultrasound innovations in abdominal radiology: techniques and clinical applications in pediatric imaging

**DOI:** 10.1007/s00261-024-04616-x

**Published:** 2024-10-16

**Authors:** Laura May Davis, Santiago Martinez-Correa, Colbey W. Freeman, Caroline Adams, Laith R. Sultan, David Q. Le, Natae Lemessa, Kassa Darge, Misun Hwang

**Affiliations:** 1 Children’s Hospital of Philadelphia, Philadelphia, PA USA; 2 University of Pennsylvania, Philadelphia, PA USA

**Keywords:** Ultrasound, Microvascular imaging, Elastography, Contrast ultrasound, Quantification

## Abstract

Contrast-enhanced ultrasound, microvascular imaging, elastography, and fat quantification have varying degrees of utility, with some applications in the pediatric setting mirroring that in adults and having unique uses when applied to children in others. This review will present novel ultrasound technologies and the clinical context in which they are applied to the pediatric abdomen. New ultrasound technologies have a broad range of applications in clinical practice and represent a powerful diagnostic tool with the potential to replace other imaging modalities, such as magnetic resonance imaging and computed tomography, in specific cases.

## Introduction

B-mode ultrasound has long been a staple of pediatric abdominal imaging as a non-invasive, affordable, and accessible test. However, the multitude of new approaches and technological developments in ultrasound have broadened the horizons of what can be imaged, and indications for ultrasound technologies in the pediatric abdomen are ever-expanding. This review will focus on the clinical indications—both current and emerging—that should prompt consideration of these novel ultrasound techniques. We will review the organ systems and pathologies that benefit from the use of (1) contrast ultrasound, (2) microvascular imaging, (3) elastography, and (4) fat quantification. As with many fields of medicine, the pediatric abdomen is not simply a smaller version of the adult’s: sonographic appearances can vary hugely depending on age, and pathologies in children include many adult diseases as well as many that are specific to pediatrics. The approach to a focal liver lesion, for example, is very different in a 1-month-old than in an 80-year-old, and the imaging approach should be sensitive to this. While ultrasound has previously been seen as a first-line screening test, the novel ultrasound technologies detailed below demonstrate its far wider applicability. These approaches rival the imaging gold standard of magnetic resonance imaging (MRI) in many instances and offer diagnostic and prognostic information that other modalities fail to convey.

## Contrast-enhanced ultrasound imaging using microbubble contrast agents

For imaging microvasculature, ultrasound contrast agents (UCAs), comprised of a phospholipid shell with an inert gas core between 1 and 10 um diameter, are intravenously injected into the patient. Due to their size, UCAs perfuse throughout the body without leaking from the vasculature, providing a blood pool marker. In comparison to tissue, UCAs exhibit a nonlinear response to acoustic waves, producing harmonics of the transmitted frequency due to an uneven expansion and compression of the microbubbles, resulting in a markedly different signal return in comparison to tissue background. As a result, nonlinear imaging techniques have been developed for contrast-specific imaging which rely on low-mechanical index (MI; below 0.3) to prevent UCA destruction and reduce tissue nonlinearity effects [1]. Furthermore, the ability to image a lesion continuously with CEUS gives it excellent temporal resolution that exceeds that of CT and MRI. By using UCAs, tissue perfusion can be both visualized and quantified, allowing for improved sensitivity and specificity to vascularity changes in real-time.

### Clinical applications of contrast-enhanced ultrasound imaging

CEUS is particularly advantageous in the pediatric population compared to other cross-sectional imaging techniques. Computed tomography (CT) utilizes ionizing radiation and MRI often requires sedation or anesthesia in the pediatric population to obtain diagnostic quality images during prolonged exam times. CEUS avoids radiation and can be performed in an awake child in a short period of time. As a result, CEUS has been increasingly used in the pediatric population to characterize lesions in the abdomen and pelvis and to assess for intra-abdominal traumatic injury in the stable patient.

**Fig. 1:**
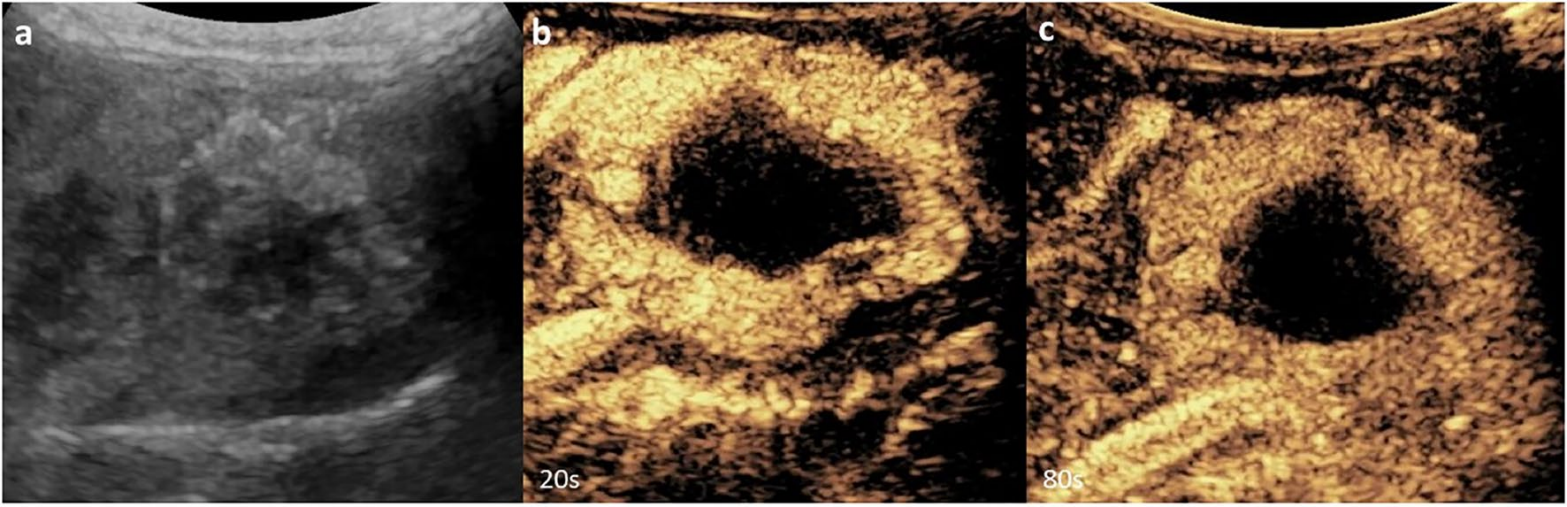
16-week-old female. Contrast ultrasound of the liver, transverse right upper quadrant scan. Post-natal evaluation of a pre-natally diagnosed congenital hemangioma showing **a** grayscale appearance of a heterogenously echogenic lesion with poorly defined borders and central hypoechogenicity; and post contrast images showing **b** arterial and **c** late venous phase enhancement, with persistent hypoenhancement centrally but no washout

**Fig. 2:**
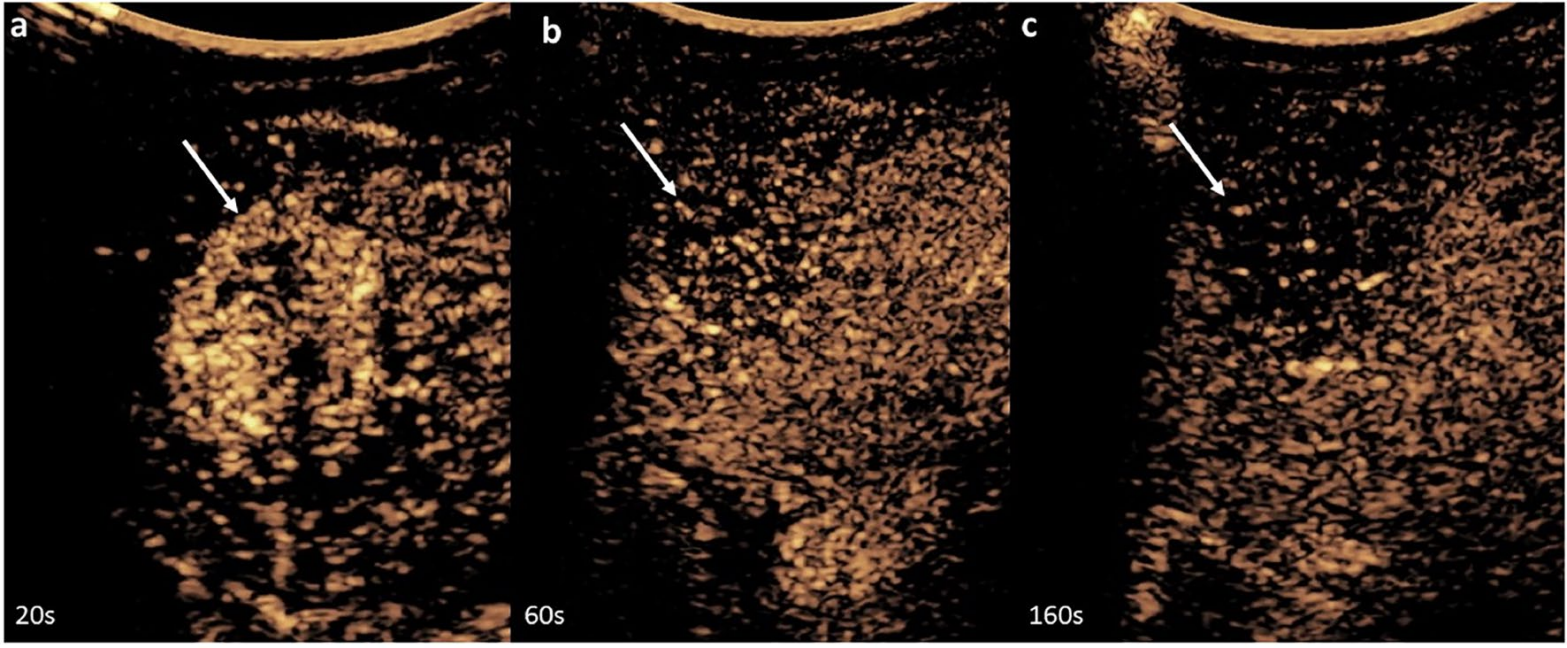
7-month-old female. CEUS study of the liver. Right upper quadrant coronal view, showing a heterogeneously hyperenhancing liver lesion with avid peripheral enhancement on arterial phase imaging **a** with washout starting at around 50 seconds as seen on **b** portal venous and **c** late venous phases in keeping with malignancy. This was biopsy-proven hepatoblastoma

**Fig. 3:**
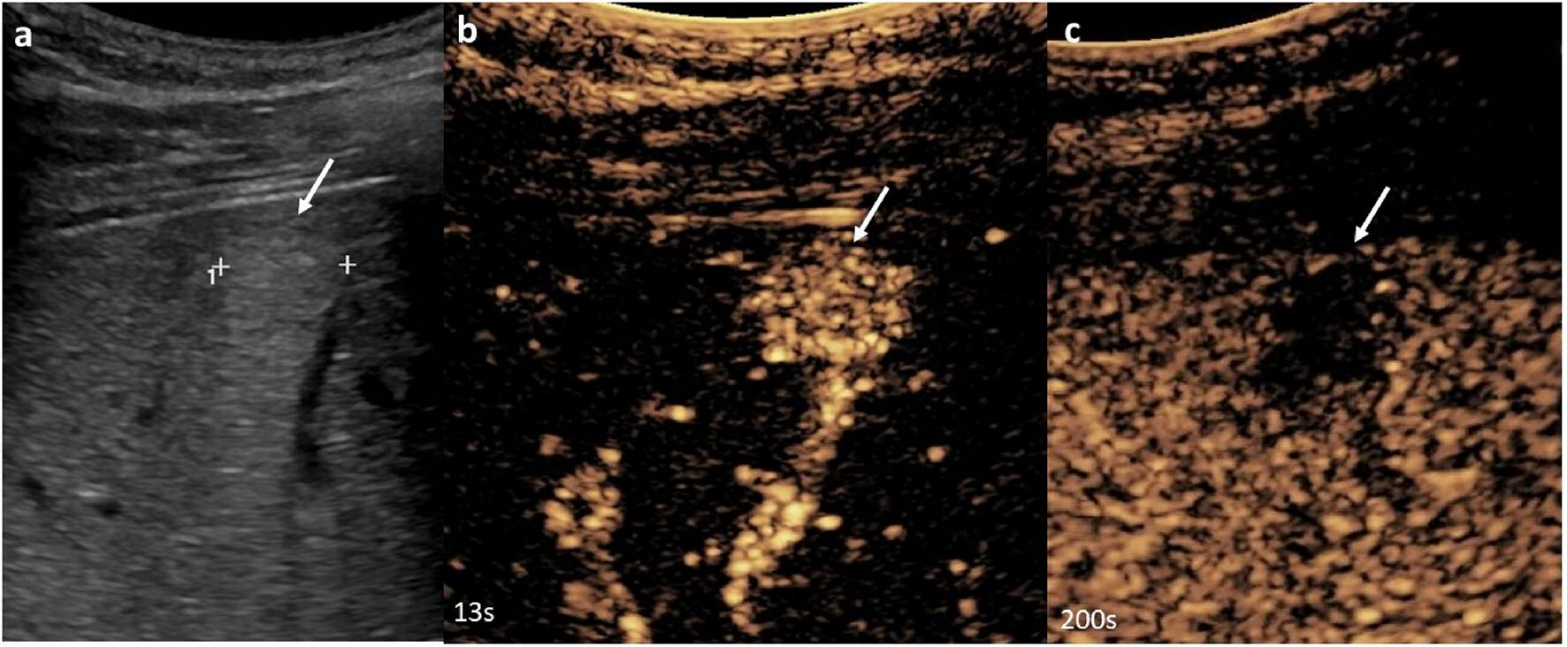
18-year-old male, background of Li Fraumeni syndrome. CEUS of the liver performed to characterize multifocal liver lesions detected on prior imaging. **a** Grayscale ultrasound showed multiple hyperechoic lesions throughout the liver, with **b** homogeneous hyperenhancement on arterial phase and washout on **c** late venous phase, in keeping with malignancy. Colonoscopy showed a sigmoid colonic mass; biopsy showed adenocarcinoma

CEUS is most frequently used to characterize hepatic lesions and to identify benign lesions that require no further work-up (i.e., avoiding unnecessary MRI/CT or biopsy). In the liver, contrast perfusion is observed during arterial, portal venous, and late phases after intravenous administration. Benign lesions such as infantile and congenital hemangioma (Fig. 1), focal nodular hyperplasia, and hepatic adenoma usually show absence of washout on late phase images, similar to their pattern of enhancement on contrast-enhanced CT and MRI (although atypical hemangiomas can show washout) (Figs. 2 and 3) [2–4]. In comparison, malignant lesions, such as hepatocellular carcinoma and hepatoblastoma, are more likely to demonstrate wash-out of UCA, typically late in HCC and earlier in hepatoblastoma [2, 3, 5]. A consensus statement released in 2020 by the American College of Radiology Pediatric LI-RADS Working Group summarizes the typical enhancement pattern and grayscale sonographic appearance of common pediatric liver lesions [6].

**Fig. 4:**
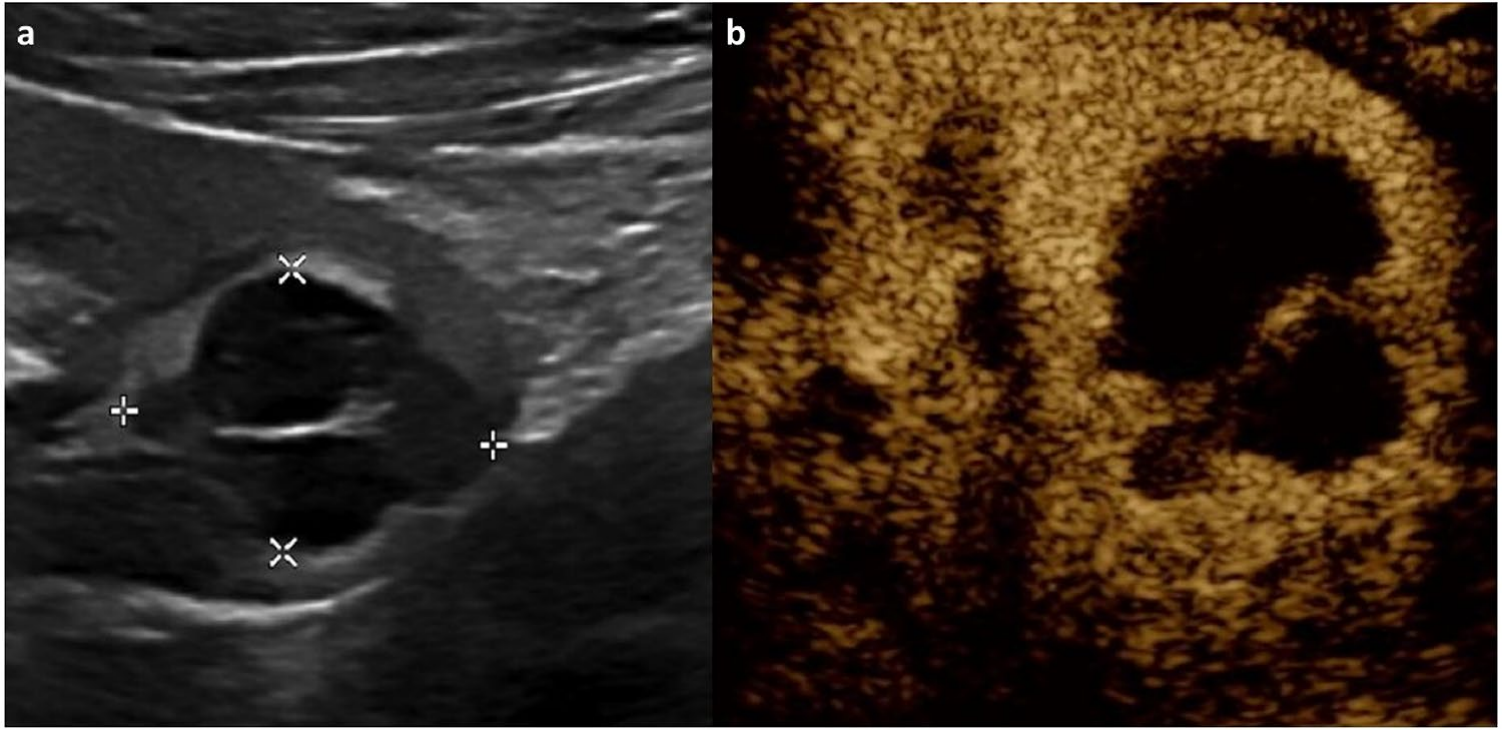
17-year-old male. CEUS study of the left kidney, transverse section. Renal CEUS performed following incidental finding of a renal cyst on CT abdomen and pelvis being performed for jaundice, showing a multiloculated lesion on grayscale (**a**) with a thin, enhancing septation on CEUS (**b**) and no solid component, graded IIF

CEUS in non-liver organs is less well-established, and is therefore less helpful for solid mass evaluation in this context. In the kidney, contrast uptake can be observed during the cortical, medullary, and late phases after intravenous administration [1]. Benign pseudo-lesions are identified (e.g., fetal lobulation, hypertrophied column of Bertin, dromedary/splenic hump) because they enhance similarly to neighboring parenchyma in all phases [7, 8]. In comparison, malignant lesions are expected to differ in enhancement from normal parenchyma in at least one phase, and lesions demonstrating such behavior warrant further investigation; when compared to histology, the combination of hyperenhancement and washout in pediatric renal lesions has 95% sensitivity and 80% specificity in detecting malignancy [9, 10]. CEUS can also be used to characterize and follow indeterminate or infectious renal lesions in children. For example, renal cystic lesions and enhancing septations are well visualized by CEUS, and subtle enhancement of septa can be appreciated with this modality (Fig. 4) [9, 11, 12]. Additionally, rim-enhancing renal abscess can be differentiated from pyelonephritis/focal nephritis, and grayscale US may then be used for follow-up to ensure resolution [7].

**Fig. 5:**
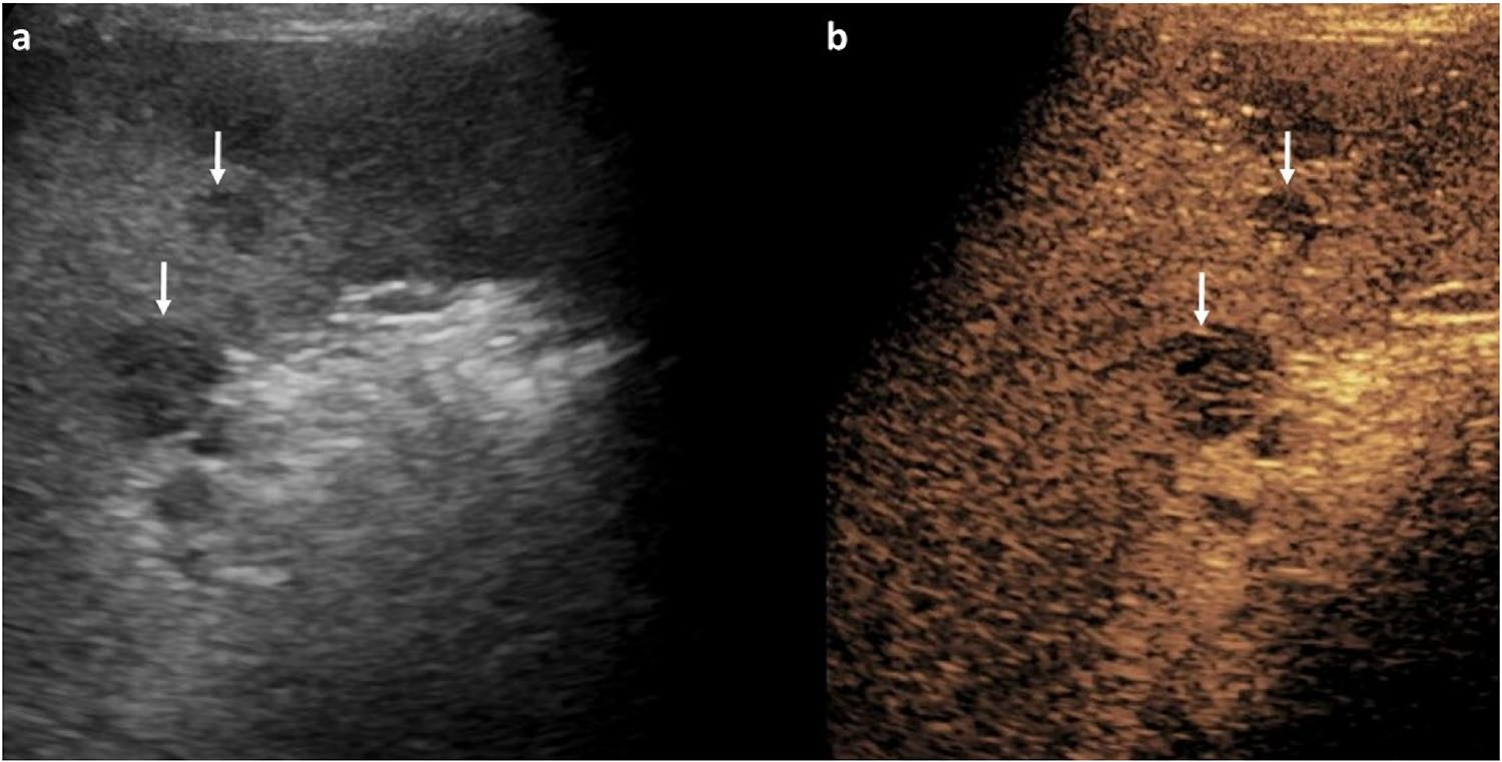
18-year-old male. Contrast ultrasound of the spleen in a patient with multiple splenic lesions, undergoing chemotherapy showing multiple hypoechoic lesions on **a** grayscale with the differential including malignant deposits or fungal dissemination; these were all hypoenhancing following **b** contrast administration, in keeping with disseminated fungal disease

In addition to the kidney and liver, CEUS has been utilized to characterize lesions in the adrenal glands, spleen, pancreas, and gonads. In the spleen, contrast uptake is observed to mimic the “zebroid” arterial pattern described in CT and MRI and has a homogeneous parenchymal phase, which can be helpful in differentiating benign lesions such as cysts, accessory splenules, slow flow venous malformations, abscess or infarct from malignant disease (Fig. 5) [13, 14]. Similarly, CEUS may be used to help distinguish between adrenal hemorrhage and neuroblastoma in the neonate [9, 15]. Pancreatic lesions, although rare in children, have also been studied. CEUS has been used to visualize both benign cystic lesions and malignant solid masses in pediatric cases, and it may be helpful in identifying acute interstitial pancreatitis from necrotizing pancreatitis, as necrotic tissue does not enhance [13]. Lastly, CEUS has been used to study lesions in the pediatric testes and can help to identify segmental infarcts, epidermoid cysts, and testicular masses [16].

Although most frequently used to characterize focal lesions in the abdomen and pelvis, CEUS can also be used to identify and follow traumatic injuries (e.g., active bleeding; liver, splenic, and renal lacerations; pancreatic pseudocyst) in the stable patient [17]. The above applications have utilized intravenous contrast; however, UCAs can be injected into the urinary bladder and CEUS performed as an adjunct or alternative to the conventional fluoroscopic voiding cystourethrogram [18, 19].

Collectively, CEUS is poised to play an increasing role in imaging of the pediatric abdomen and pelvis. However, pediatric applications of CEUS are understudied compared to their adult counterparts, and more data is needed.

### Quantitative methods and applications for contrast-enhanced ultrasound imaging

As UCAs perfuse an organ, the microbubble uptake in the tissues is directly correlated with vascularity. Most published CEUS studies have relied upon qualitative assessment of enhancement over time. However, bubble influx can be quantified by recording the image intensity as a function of time (time-intensity curve or TIC) [20]. Quantification can be performed in two ways: one through the injection of UCAs as a bolus and watching the influx and efflux of bubbles at a target site (wash-in/wash-out), and the other during a steady-state infusion where bubbles in the imaging frame are destroyed through a high-pressure sequence and are imaged as bubbles re-perfuse into the tissues. The wash-in/wash-out analysis is typically performed in the clinic as it can be applied without the use of an infusion pump, reducing administration complexities. Burst replenishment methods are limited to use in the research settings and have been discussed in detail elsewhere [21]. Some metrics of interest for analyzing the TICs are the peak enhancement (maximum intensity), wash-in area under the curve, time to peak, wash-in rate, wash-out area under the curve, and wash-out rate. The TIC analyses can be performed for both wash-in/wash-out and destruction-reperfusion methods through vendor-specific on-system software or through offline applications such as VueBox (Bracco, Milan, Italy).

**Fig. 6:**
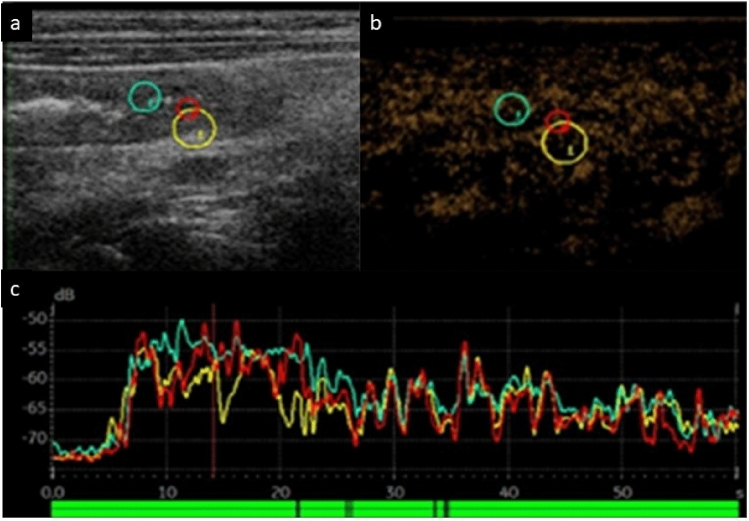
2-year-old male. Quantitative CEUS time-intensity curve (bolus injection) of the descending colon shows peak enhancement of 20 dB from baseline and early arterial phase transmural hyperenhancement, compatible with active inflammation. Grayscale image on the left (**a**), contrast on the right (**b**), with a curve of dB plotted against time on the bottom (**c**) showing the difference of 20 dB compared to baseline at 10–15 s

CEUS has been employed in pediatric populations, especially for assessing renal perfusion, Crohn’s disease (Fig. 6), liver lesions, and antiangiogenic therapeutic efficacy in solid tumors [22–28]. In these applications, CEUS has shown increased sensitivity and specificity compared to conventional Doppler flow imaging, providing improved visualization of microvasculature and tissue perfusion. An example of CEUS applied for assessing very early onset inflammatory bowel disease is shown in Fig. 6 where the peak enhancement and time to peak indicate increased inflammation. Advanced particle tracking techniques, used in super-resolution imaging, allow high spatial resolution imaging to the level of the microvessels, overcoming the spatial resolution limit of conventional ultrasound. Velocity mapping using microbubbles allows a greater understanding of blood flow within these tiny vessels and has potential to be employed as a valuable biomarker of organ health [29].

Quantitative DCEUS shows great promise for improving diagnostic accuracy over qualitative reading techniques. However, variability in application and acquisition such as microbubble administration, motion, and accelerated immune system clearance of UCAs need to be better understood to allow for greater reproducibility and ease of use [30, 31].

## Microvascular imaging (MVI)

Microvascular imaging (MVI) ultrasound is a Doppler-based ultrasound technology with multiple potential applications in the abdomen and pelvis. Conventional color Doppler uses a unidimensional wall filter to remove noise and clutter to identify vascular signal, but this has the negative effect of removing slow and low-volume flow from the images. MVI uses a more complicated multidimensional wall filter combined with motion correction to achieve high frame rate imaging that can detect slow and low-volume flow, facilitating visualization of the microvasculature without contrast agents. Although the processing methods are proprietary and vendor-specific, several options have been made available to the clinic such as Superb Microvascular Imaging (Canon Medical, Otawar, Japan), Microvascular Imaging (GE Healthcare, Milwaukee, WI, USA), Microflow Imaging (Philips Healthcare, Best, Netherlands), Slow Flow (Siemens Healthineers, Richmond, VA, USA), and MV-Flow (Samsung Medison Co., Ltd., Seoul, Korea). Compared to traditional color Doppler imaging methods, MVI provides greater vessel detail at improved frame rates, although direct comparison between MVI and spectral waveform are limited.

### MVI Quantification methods

Quantification techniques for MVI are still in their infancy, but methods that have been developed for conventional color and power Doppler techniques are potentially more capable given their improved resolution and definition of the microvasculature which was previously difficult to visualize. One such use—initially developed in the setting of breast cancer, with semi-quantitative grading of flow (from 0 (no flow) to 3 (marked vascularity) [32]—has been applied to multiple clinical applications in pediatrics. In the assessment of mesenteric lymphadenitis, the addition of this grading system showed improved sensitivity and specificity compared to conventional grayscale and color Doppler ultrasound imaging [33].

Hemodynamic parameters such as resistive index, pulsatility index, and vascularity index can be calculated from MVI data through the blood flow velocities in the vessels over time. This has commonly been performed in conventional Doppler imaging for assessing vascular functions in tissues such as the bladder wall, kidney, and umbilical vessels [34–36]. The vascularity index has been successfully used for improved detection of kidney dysfunction and renal fibrosis as well as acute cystitis in children [34, 37].

### Applications

As a relatively new technology, the use of MVI within abdominopelvic imaging is an emerging topic within the literature and clinic. Its applications vary by organ but include evaluation of neoplasm, organ perfusion, and blood flow.

### Liver

**Fig. 7:**
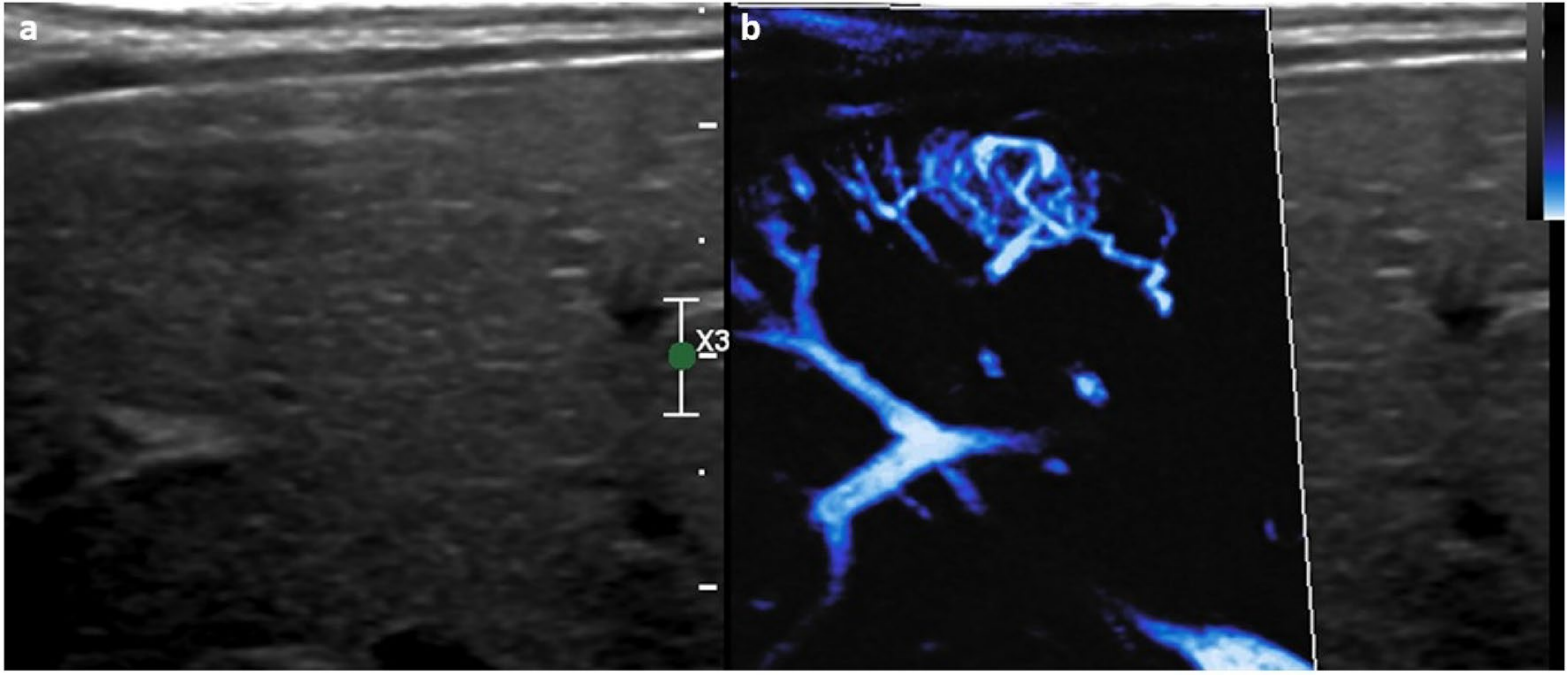
10-month-old male, background of Beckwith-Wiedemann syndrome. **a** Grayscale image, transverse plane, of a poorly-defined, hypoechoic lesion. **b** MVI demonstrates peripheral vascularity in a centripetal pattern in keeping with an infantile hemangioma that remained stable stable of subsequent imaging

The characterization of hepatic lesions is a potential application of MVI. Contrast agents used in CEUS, CT, and MRI are excellent for diagnostically differentiating hepatic lesions but require invasive administration of intravenous contrast. MVI offers an alternative to contrast-enhanced imaging for lesion characterization, although its sensitivity for vascularity is lower than CEUS, showing a 73.75% concordance in one study [38]. The most common benign hepatic lesions are hemangiomas. Approximately two-thirds of hepatic hemangiomas demonstrate flow on MVI, with the most common patterns being “nodular rim,” “strip rim,” “spotty dot-like,” and “diffuse dot-like” [39–43]. Therefore, MVI may obviate the need for contrast when evaluating these lesions in many instances (Fig. 7).

Focal nodular hyperplasia is the second most common benign hepatic lesion. Histopathologically, focal nodular hyperplasia comprises fibrous tissue and vessels radiating from a central scar. Its appearance on MVI is reflective of this structure with radiating or spoke-like patterns of vascularity, and can be detected on MVI with greater sensitivity than color Doppler [39, 41, 44]. Adenomas, the third most common benign hepatic lesion, may demonstrate “diffuse honeycomb” vascularity [40].

MVI may demonstrate certain vascularity patterns in malignant lesions. He et al. [40] described several cases of “strip rim” vascularity in metastases with one example of a lesion with a “thick rim”. Hepatocellular carcinoma may show diffuse honeycomb or non-specific vascularity, predominantly peripheral flow, or a combination of peripheral and central flow [40, 42]. Vascularity on MVI and enhancement on CEUS have strong correlation in the evaluation of liver metastases [45]. MVI can assist with detecting residual disease in hepatic lesions following transarterial chemoembolization [46]. Nevertheless, findings on MVI may not be sufficiently specific in these scenarios to eliminate the need for contrast or other modalities and further work is warranted.

The evaluation of organ transplant complications is another potential application of MVI. Thrombosis is a potentially devastating complication of organ transplantation, and conventional Doppler is not as sensitive to the detection of slow flow within vessels as MVI. In a study evaluating the visibility of the hepatic artery after pediatric hepatic transplantation, the authors found that most of the hepatic artery was visible in significantly more cases and with less venous signal contamination with MVI than conventional Doppler [47]. Tokodai et al. [48] reported a case in which the splenic vein was monitored using conventional Doppler and MVI for several weeks after adult pancreas transplantation, and MVI repeatedly demonstrated flow within the vein where conventional Doppler did not.

### Gallbladder

MVI may similarly lend itself to the evaluation of neoplastic processes in the gallbladder. However, current research is limited, and in one study, MVI only demonstrated usefulness when contrast was also employed [49]. Without contrast, the quality of MVI was insufficient to assist with diagnosis of gallbladder lesions [49].

**Fig. 8:**
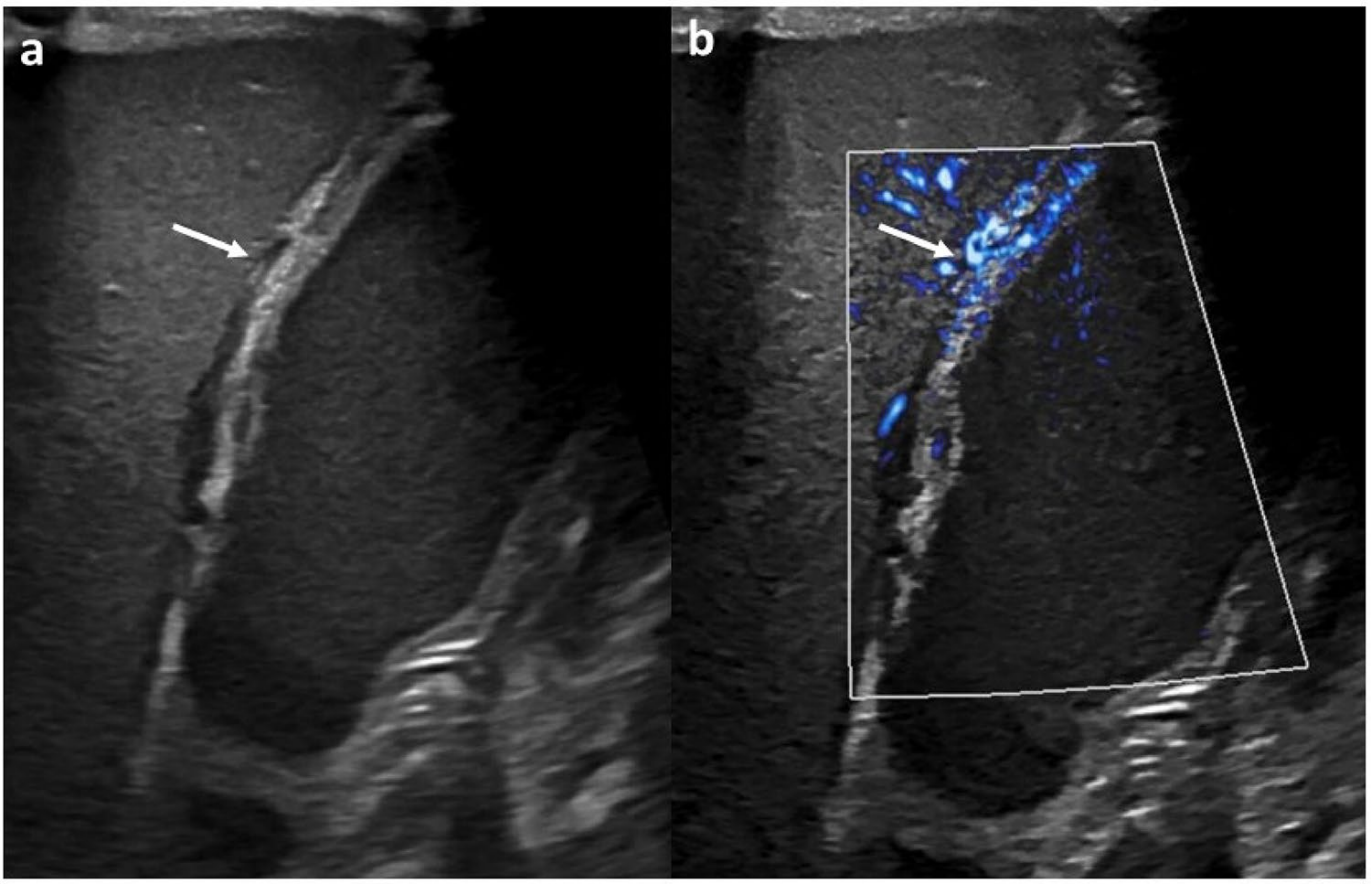
17-year-old female with right upper quadrant pain in the setting of sepsis. **a** Grayscale showed sludge within the gall bladder and wall thickening (arrow), with **b** MVI confirming that there was hypervascularity (arrow), in keeping with acute cholecystitis, requiring percutaneous cholecystostomy

MVI’s sensitivity for flow is useful in the evaluation of infectious and inflammatory processes of the gallbladder (Fig. 8). MVI increases sensitivity for detecting acute cholecystitis compared to conventional Doppler [50]. The intensity of signal on MVI may be correlated with cholecystitis severity [51]. By detecting perfusion defects in the gallbladder wall, MVI also increases detection of gallbladder perforation [52].

### Kidney

**Fig. 9:**
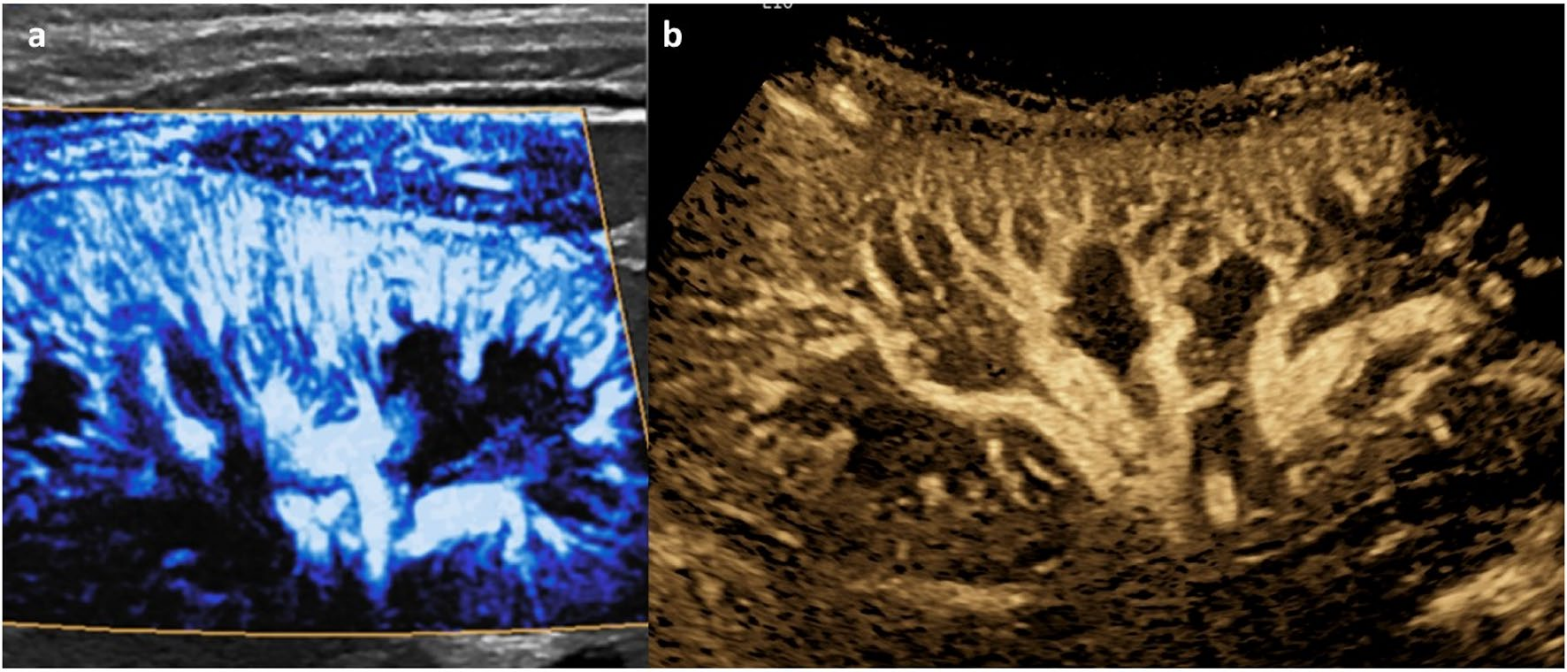
3-year-old male. Microvascular imaging of the kidney in sagittal plane clearly delineating the renal parenchymal **a** Philips, and **b** using GE scanners

As with hepatic lesions, MVI can be employed to evaluate renal lesions. MVI is more sensitive and accurate for differentiation of malignant and benign renal lesions than conventional color Doppler but less sensitive than CEUS for detection of vascularity [53]. When compared to CEUS as the reference standard, MVI has greater than 60% sensitivity and specificity for detection of flow within renal masses [54]. However, the evidence base is in the adult population with little work currently addressing renal masses in children evaluated with MVI. Where MVI has been shown useful in children is in the setting of perfusion imaging; MVI is superior to conventional Doppler for assessing renal cortical vasculature, detecting both a greater density of microvessels and more of their course toward the cortex (Fig. 9) [55]. In the setting of urinary tract infection, MVI can be used to clarify regions of hypoechoic renal parenchyma, allowing the identification of focal lobar nephronia, an intermediate stage between pyelonephritis and renal abscess (Fig. 11). The degree of chronic kidney disease and fibrosis present is negatively correlated with renal vascularity. In children evaluated with MVI, there was a strong negative correlation between an MVI derived vascularity index and progression of hydronephrosis related renal parenchymal disease [56].

**Fig. 10:**
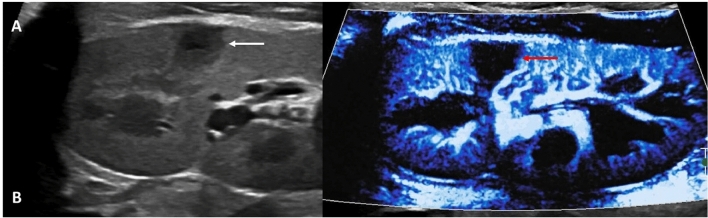
17-year-old male. Images of the left kidney in the setting of fever and dysuria. The grayscale image (A, white arrow) shows an area of hypoechogenicity, suspicious for focal nephronia, with (B) MVI demonstrating lack of flow here and showing it to be more extensive than grayscale suggested (red arrow) and confirming the diagnosis, thus obviating the need for further imaging to characterize

MVI can also detect inflammatory and infectious lesions in the kidney. Conventionally, pyelonephritis is associated with perfusion defects on Doppler ultrasound, contrast-enhanced CT, and technetium-99 m dimercaptosuccinic acid scans. MVI was found to be superior to color Doppler for the detection of hypoperfusion associated with acute pyelonephritis and may aid in confirmation of grayscale findings (Fig. 10) [57].

As with the liver, MVI may prove useful in the evaluation of renal allografts. Cortical perfusion is diminished in dysfunctional renal allografts. MVI can more easily detect renal cortical perfusion than color and power Doppler, facilitating imaging evaluation of renal transplants. In children and adults with renal transplant, MVI had higher specificity than conventional Doppler for the detection of chronic allograft damage [58].

### Testes

MVI’s ability to detect low and slow flow is useful in the testes, where MVI is superior to conventional Doppler for detection of testicular blood flow. This is particularly useful in pediatric patients with small testicular volume for whom confirmation of testicular torsion by imaging would otherwise not be possible [59]. MVI is also capable of differentiating subtle differences in perfusion of undescended testicles that are otherwise not evident on conventional Doppler [60].

### Bowel

MVI may be employed to evaluate microvascular perfusion in the bowel as well. MVI has been used to demonstrate increased bowel wall perfusion in adults with inflammatory bowel disease, where inflammation results in vascular proliferation within the bowel wall and adjacent mesentery [63]. The degree of microvascular perfusion is correlated with severity of ulcerative colitis on endoscopy [63]. The use of MVI for evaluation of the bowel in children is less well established, but it has been used in children to demonstrate similar inflammatory entities, such as enteritis [61, 62]. Although research is needed, MVI may assist in diagnosis of inflammatory conditions where color and power Doppler are currently utilized, such as appendicitis and diverticulitis. MVI may also serve a supplemental role in the evaluation of bowel and mesenteric masses.

### Spleen

There is a gap in the literature regarding the use of MVI for the evaluation of splenic lesions, but we have used MVI clinically for this purpose, demonstrating no flow associated with a cystic lesion (Fig. 13). Potential uses include assessment of splenic vascular malformations with slow flow, allowing detailed vascular mapping and the ability to assess vessel involution following treatment. MVI may also be used for evaluation of splenic vasculature, with one case report demonstrating superior visualization of flow within a splenic pseudoaneurysm in an adult over CT and color Doppler [63].

### Lymph nodes

**Fig. 11:**
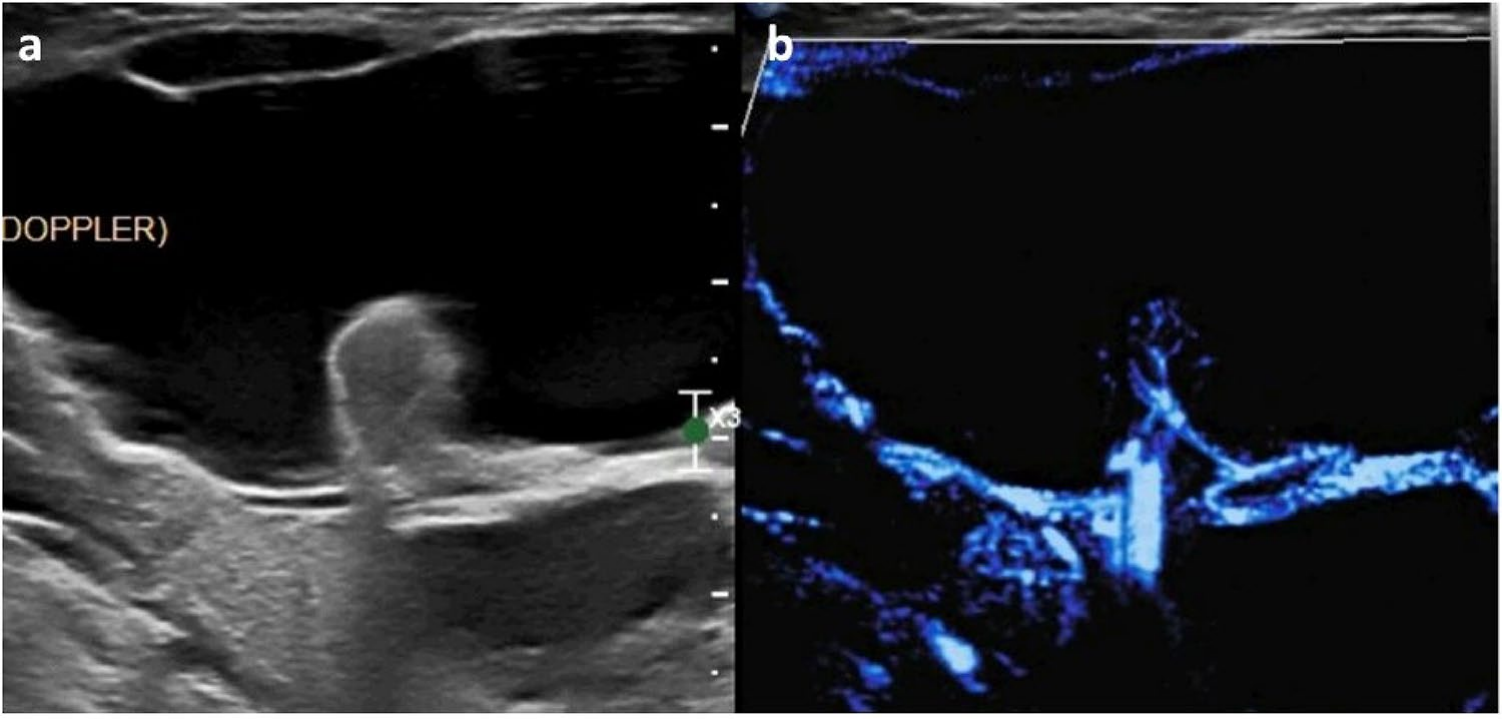
12-year-old boy. MVI as part of ultrasound for characterization of a neck mass that presented with left sided facial swelling. **a** Grayscale ultrasound demonstrated a large, predominantly cystic lesion with a solid central region, concerning for a solid nodule. **b** MVI showed hilar vessels, characteristic of a lymph node, most in keeping with a lymphatic malformation with a central lymph node

Abnormal lymph nodes often pose a diagnostic challenge and differentiating reactive/inflammatory from neoplastic ones can have important consequences for guiding treatment and prognostication. Vascular patterns within a lymph node on MVI may facilitate differentiation between these entities (Fig. 11). In studies comparing metastatic and tuberculous cervical lymph nodes, greater and more central vascularity was associated with metastatic nodes [64, 65]. The specific pattern of vascularity may depend on the neoplastic cells’ route of entry to the node, whether peripheral or through central hilar vessels [65]. For example, in papillary thyroid cancer, there is increased angiogenesis peripherally in metastatic lymph nodes that helps differentiate them from benign nodes on MVI [66]. MVI also has utility in demonstrating normal vascular morphology in non-pathologic lymph nodes, such as in the setting of mesenteric adenitis where grayscale and MVI had sensitivity and specificity (82%, 79% respectively) that exceeded grayscale with color Doppler flow imaging (74% sensitivity ad 75% specify) [33].

## Elastography

Elastography refers to a non-invasive imaging technique for measuring the internal deformation or stiffness changes in tissues that result from the application of mechanical stress [67]. This technology has been developed for both MRI and ultrasound. However, due to the limitations of MRI discussed above, the clinical applications of ultrasound elastography are rapidly expanding. Ultrasound elastography is similar in principle to manual palpation, the physical examination technique by which the mechanical properties of tissue are detected. Such changes in tissue mechanics—resulting in increased or decreased stiffness—usually occur with different disease processes [68]. Ultrasound elastography has been widely utilized in the adult population, most commonly to assess hepatic stiffness in the context of fibrotic liver disease [69–71]. However, despite widespread utilization in adults, there remains a paucity of evidence around the use and effectiveness of elastography in the pediatric population.

### Technical aspects of ultrasound elastography

Elasticity refers to a tissue’s tendency to resist deformation when a force is applied yet recovers its original shape upon that force being removed. Stiffness is inversely proportional to elasticity—the higher the stiffness, the more resistant a tissue is to deformation; stiffer tissue deforms less when force is applied compared to more elastic tissue [72].

Sonographic elastography can be performed in two ways, depending on the nature of the applied external mechanical stimulus. In strain-based elastography (static method), the force applied to the tissue is exogenous probe pressure or endogenous mechanical forces (e.g., cardiovascular/respiratory pulsation). In comparison, shear-wave elastography (SWE; dynamic method) refers to scenarios where the force is induced by the imaging system in the form of a high-pressure acoustic wave known as the acoustic radiation force impulse (ARFI). Acquisition protocols for ARFI SWE have been extensively detailed in the Society of Radiologists in Ultrasound elastography consensus document [71].

### Clinical applications

#### Liver

Ultrasound elastography of the liver is a well-established technique in adults, where it provides a surrogate technique for biopsy in the diagnosis of hepatic fibrosis [73]. It shows promise for non-invasively assessing liver fibrosis and chronic liver disease in children. It is typically performed with the patient supine, via an intercostal approach to visualize the right hepatic lobe and the probe held perpendicular to the skin surface [74–76]. The ROI box is aligned perpendicular to the ultrasound beam to minimize shear wave reflections. Quantitative shear wave speed values are provided in meters per second (m/s) or converted to a liver stiffness measurement in kilopascals (kPa) [67, 77]. At least 10 acquisitions are recommended, with the average value used for assessment [76].

There are limitations to the use of hepatic elastography in children. Some are child-specific, such as inability to breath hold or remain motionless which results in non-diagnostic images. Age is equally important; the younger the child, the greater difficulty they may have following breath-hold instructions, with up to 27% of studies having invalid results in this age group [74]. Similarly, in the distressed, crying patient images are likely to be non-diagnostic; feeding can help with settling [78]. Others are also encountered in the adult population, such as alterations in hepatic blood flow in the post-prandial state or following alteration of hepatic venous drainage, as in the population with post-Fontan liver disease [77]. Factors like inflammation can confound liver stiffness values, so elastography may need to be interpreted in context of other lab and imaging findings [79]. In addition, stiffness as assessed by ultrasound elastography has been shown to be influenced by age and body mass index [80, 81].

**Fig. 12:**
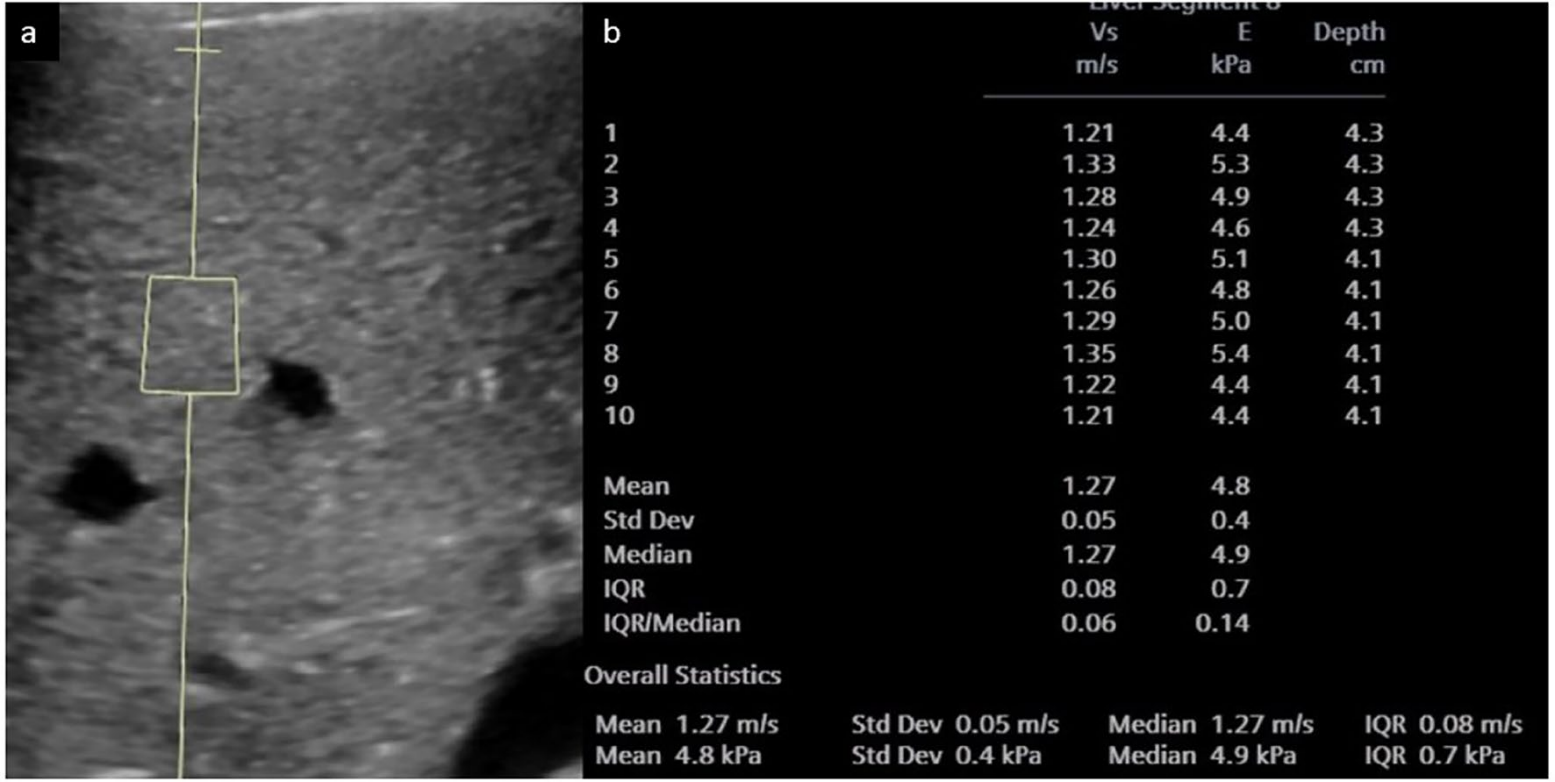
17-year-old female with argininosuccinate lyase deficiency and elevated liver enzymes. **a** Grayscale of the liver with elastography region of interest. **b** Summary following 10 elastography readings. Mean stiffness was increased, in keeping with hepatic parenchymal disease

Elastography has demonstrated good accuracy for diagnosing cirrhosis and distinguishing healthy liver tissue from fibrotic tissue [74, 79, 82]. Studies have found SWE values correlate significantly with degree of fibrosis on liver biopsy [79, 82]. Potential pediatric applications include monitoring conditions like cystic fibrosis-related liver disease, biliary atresia and those post-Kasai procedure, Wilson’s disease, other metabolic abnormalities (Fig. 12) and fibrosis after liver transplantation [72, 74, 79, 83]. In the evaluation of hepatic fibrosis in children and adolescents, a meta-analysis of 550 patients found that elastography has a sensitivity of 81% and a specificity of 91% in the detection of fibrosis [84]. Other studies have derived cutoff values for liver stiffness measurements for the diagnosis of fibrosis in various disease states [77, 84–87]. Elastography can also be used to predict portal hypertension; a meta-analysis by Kim et al. [88] found elastography (all types pooled) had 90% sensitivity and 79% specificity in predicting the presence of portal hypertension.

While early study results are promising, several questions remain regarding optimal elastography protocols and techniques for pediatric liver disease. Most data are derived from pSWE and 2D-SWE; less research has examined transient elastography in children. Available studies use varying elastography systems and acquisition protocols. This heterogeneity, along with diverse study populations, has yielded a wide range of proposed normative liver stiffness values and cutoffs to diagnose fibrosis stages across different etiologies [89, 90].

### Pancreas

Ultrasound elastography of the pancreas shows promise for screening and monitoring various conditions affecting the pancreatic tissue [74]. Acquisition of these images is limited by the pancreas’s retroperitoneal location and tendency to be obscured by bowel gas, a limitation that also affects conventional pancreatic ultrasound. However, an endoscopic approach has been suggested as a means to overcome these limitations, albeit not an approach that is accessible in all institutions [91]. Normal pancreatic shear wave speed values have been established in healthy children using 2D-SWE, with a study of 120 children finding that the median pancreatic shear wave speed was 1.31 m/s (IQR 1.21–1.40 m/s, Canon Aplio i800 system, i8CX1 transducer) [92]. Shear wave speed did not vary significantly between pancreatic regions but was positively associated with age, female gender, number of hours fasting, depth of measurement, and liver shear wave speed.

In pathological conditions, studies have demonstrated significantly lower shear wave speeds in children with cystic fibrosis (0.85–1.03 m/s, ACUSON S3000 scanner, Siemens Healthcare, Erlangen, Germany) and higher speeds in some children with type 1 diabetes (up to 7.10 m/s, using LOGIQ P9 GE ultrasound system, GE Healthcare, Milwaukee, WI) compared to controls [93, 94]. Shear wave speeds correlate with duration of type 1 diabetes and markers of diabetes control and complications, with early-stage type 1 diabetes being associated with sub-normal pancreatic stiffness and long-standing type 1 diabetes having higher than normal pancreatic stiffness. An additional association is seen between high pancreatic stiffness and complications of type 1 diabetes, including hypoglycemic episodes, neuropathy, and nephropathy [93]. Furthermore, children with Wilson's disease demonstrated reduced pancreatic shear wave speed despite normal appearances on standard ultrasound, indicating subclinical copper-related injury [83, 91]. Studies in adults have shown diffusely increased stiffness in the setting of autoimmune pancreatitis and in isolated pancreatic tuberculosis [95, 96]. Overall, 2D-SWE provides a sensitive tool for evaluating pancreatic pathology in children across a variety of pancreatic disorders.

### Spleen

Spleen ultrasound elastography examination should be performed with the patient in supine position with the left arm in maximal abduction and the transducer in the left intercostal space to allow an adequate acoustic window [67]. Some authors have aimed to establish normal values for spleen stiffness using ARFI imaging in healthy children [97, 98]. Cañas et al. found mean shear wave velocities of 2.17 m/s with a convex probe and 2.15 m/s with a linear probe in 60 children (ACUSON 2000 ultrasound machine, Siemens Medical Solutions, Mountain View, CA), while Hanquinet et al. reported a median velocity of 2.43 m/s using a curved probe in 102 children (Acuson Ultrasound System, S3000 platform; Siemens Healthineers, Erlangen, Germany) [97, 98].

**Fig. 13:**
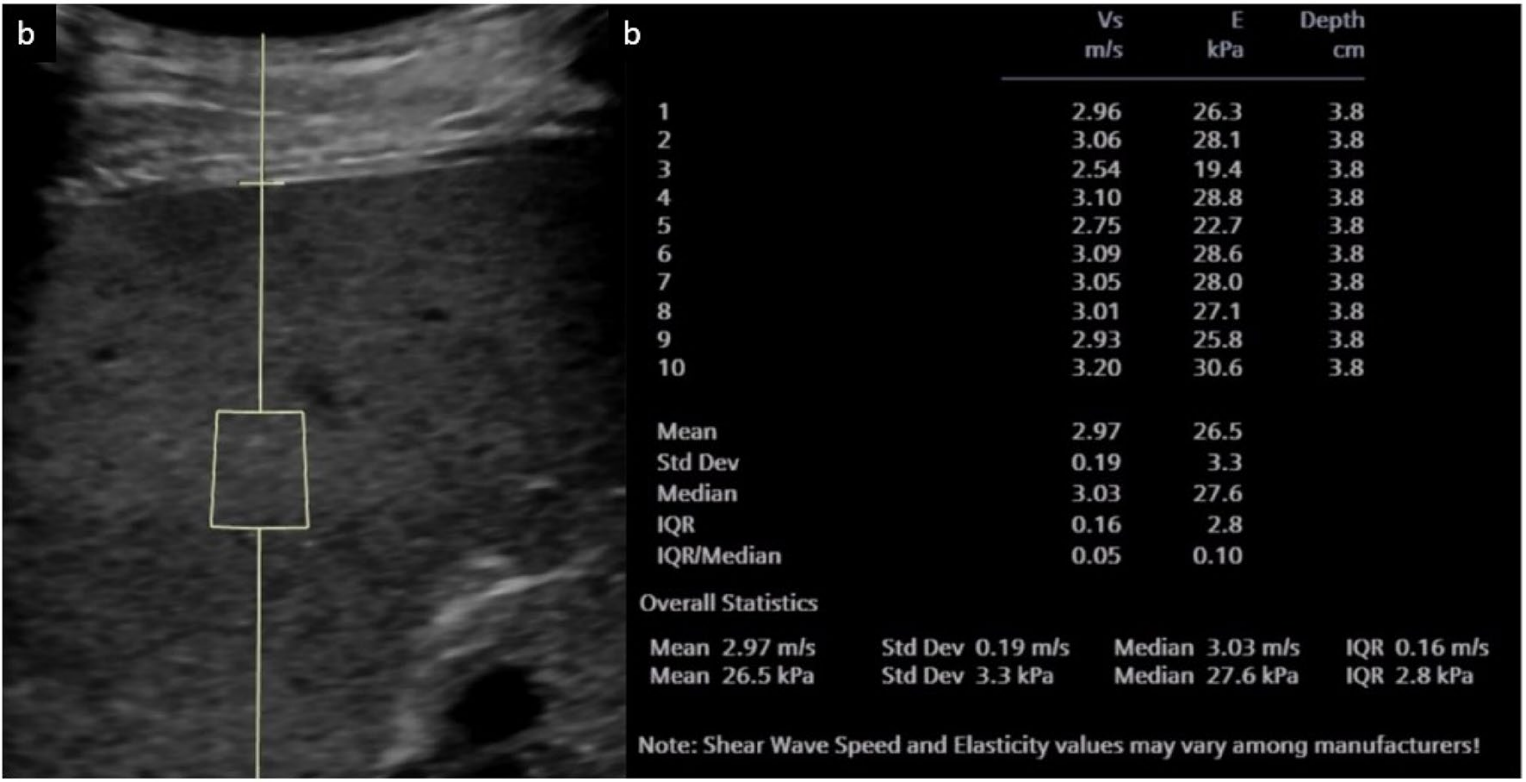
15-year-old male. Splenic elastography. Prior Fontan procedure. **a** Left upper quadrant ultrasound of the spleen with shear wave elastography acquisitions. **b** Results of the SWE, with 10 readings acquired, show increased splenic stiffness, suggestive of splenic parenchymal disease

Compared to these normal spleen stiffness values, higher stiffness has been found in patients with splenomegaly, esophageal varices, and history of variceal hemorrhage [99]. Splenic stiffness alters in the context of liver disease and provides a means of assessing portal hypertension [74]. It also allows a means of assessing patients following transjugular intrahepatic portosystemic shunt insertion [74]. Additionally, Uchida et al. [100] found that the degree of splenic stiffness was predictive of the severity of portal hypertension in patients with biliary atresia treated with Kasai procedure. Tomita et al. [101] evaluated the diagnostic value of spleen stiffness measurements by ARFI imaging in diagnosing venous complications after pediatric living donor liver transplantation, finding significantly higher median spleen stiffness values in patients with venous complications, with 100% sensitivity and 78.9% specificity (using ACUSON S2000 system, Mochida Siemens Medical Systems, Tokyo, Japan). In patients undergoing follow-up following Fontan procedure, stiffness in the spleen was shown to be significantly increased compared to healthy controls, and is a good noninvasive marker for portal hypertension and the congested hepatopathy associated with this procedure (Fig. 13) [102].

### Kidney

The kidneys’ retroperitoneal location and orientation make ultrasound elastography difficult. When imaging is being attempted, prone patient positioning allows visualization in the long axis of the kidney so that the ROI encompasses both cortex and medulla [67]. Renal ultrasound elastography shows increased stiffness in children and young adults with hypertension and chronic kidney disease, with a greater increase in the context of hypertension and further elevations when groups were subdivided by elevated body mass index [103]. In contrast, Sağlam et al. [104] compared kidney shear wave velocities between children with type 1 diabetes and healthy controls and found no significant difference in velocities between the two groups. Another potential use of ultrasound elastography is the evaluation of glomerulonephritides. For example, one prospective study of 30 children with biopsy-proven acute glomerulonephritis and 30 healthy children found that mean kidney elastography values evaluated by ARFI were significantly higher in the disease group, indicating its potential as an auxiliary method for early diagnosis of acute glomerulonephritis [105].

### Bladder

Elastography can non-invasively evaluate bladder wall stiffness and indirectly measure bladder pressure [72]. Sturm and colleagues found that patients with noncompliant bladders may have increased mean shear wave speed, reflecting increased tissue stiffness corresponding to elevated bladder pressures [106]. This application is promising since measuring bladder pressure typically requires bladder catheterization, an invasive procedure that carries the risk of infection and other complications. However, work by Calle-Toro et al. [107] suggests that this approach does not offer clinical utility at lower bladder pressures. Additionally, elastography could help characterize detrusor smooth muscle stiffness, a feature not appreciable on conventional ultrasound. This feature can be present in children with acute cystitis [108]. However, larger pediatric studies are still needed to better determine diagnostic performance and establish standardized protocols before clinical implementation.

## Fat quantification

**Fig. 14:**
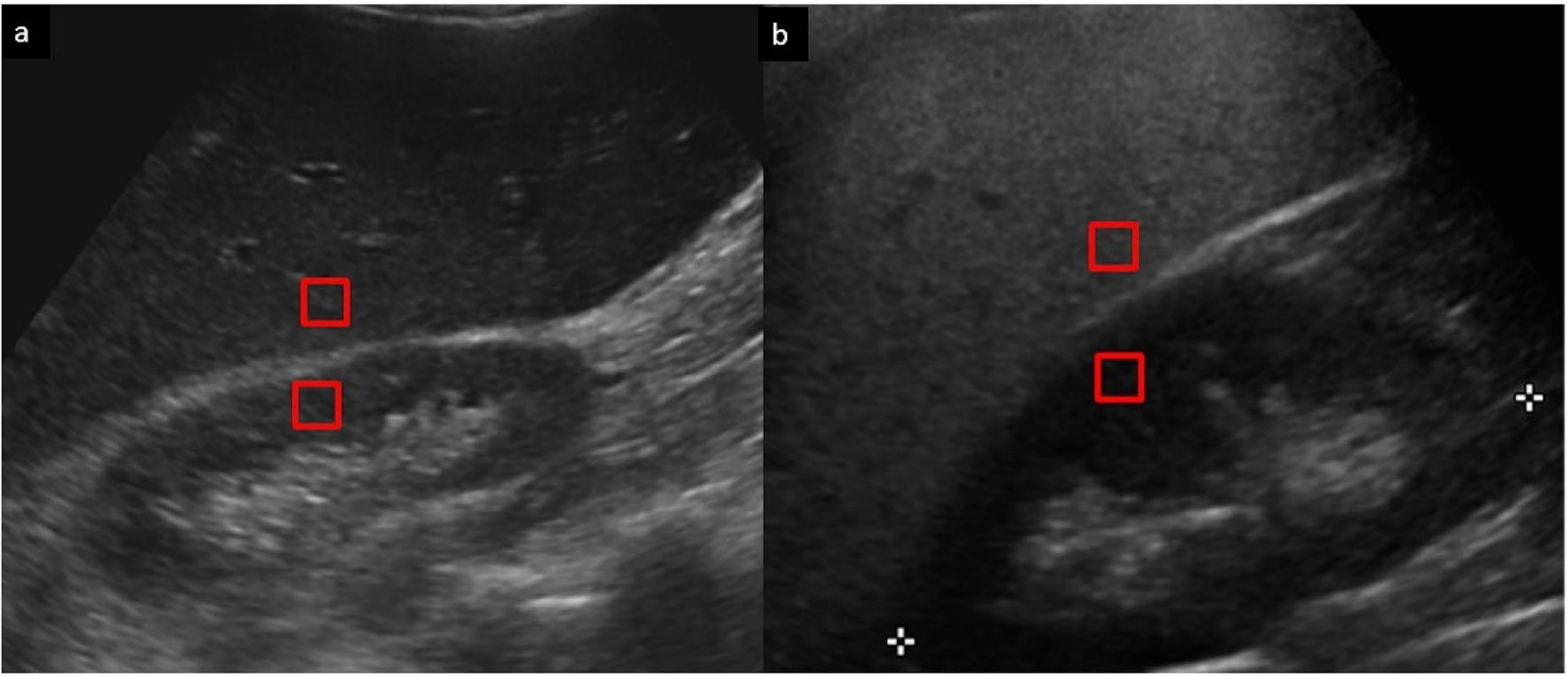
Grayscale images encompassing the liver and right kidney in sagittal plane. Regions of interest have been drawn over both to allow calculation of the hepatorenal index. **a** A normal liver with an HRI of 1.01; **b** fatty liver with an HRI of 3.2

A few approaches using ultrasound exist for assessment of fat, and so far, these have focused on hepatic pathologies. Hepatic fat content can be assessed qualitatively using B-mode ultrasound. Features of fatty infiltration include loss of periportal echogenicity, posterior acoustic drop-out, and increased echogenicity of the hepatic parenchyma [109]. These imaging features are excellent at detecting advanced steatosis when over 20% of hepatocytes are affected but have low sensitivity in the detection of mild disease. A related but more quantitative approach is the generation of a ratio of brightness of the organ of interest to another organ of known echogenicity to estimate fat content. For example, the hepatorenal index (HRI), which is specific to the liver, is derived by drawing regions of interest on an ultrasound image of the liver and right kidney in the same frame and calculating a ratio of brightness between the two (Fig. 14) [110].

A more generally applicable approach is the controlled attenuation parameter method, which allows quantification of steatosis by measuring the amount of ultrasonic attenuation in the liver (in decibels per meter), derived from analysis of ultrasonic signals arising from elastography devices. This does not allow co-acquisition of an anatomical image [111, 112]. In contrast, ultrasound attenuation calculates an attenuation coefficient based on signal loss that occurs as a result of scatter, absorption, diffusion, and reflection [113].

### Clinical applications of ultrasound fat quantification in pediatrics

#### Liver

The most clinically relevant application of fat quantification is in the setting of obesity in children and adolescents [114]. Obesity has many associated long-term complications, including hypertension, insulin resistance, type 2 diabetes mellitus, obstructive sleep apnea, and metabolic dysfunction associated steatotic liver disease (MASLD) [115]. MASLD, an accumulation of macrovesicular fat in the absence of any other cause of hepatic injury such as infection, develops on a spectrum from early fat deposition to cirrhosis requiring liver transplant [116]. It can lead to hepatocellular carcinoma and represents the most common cause of liver transplant in the United States [116]. The most recent estimates of MASLD prevalence are between 5 and 10% [116]. The gold standard for diagnosis of hepatic steatosis is a biopsy; however, this is invasive and not appropriate for screening due to the risks involved [114]. Semi-quantitative approaches that derive a liver score based on attributes including brightness, diaphragm attenuation, and vessel blurring had a sensitivity of 78% and specificity of 85% to detect NAFLD, with good agreement with MR spectroscopy [109]. HRI was found in one study in an adult population to have a cutoff value of 2.01, sensitivity of 62.5%, and specificity of 95.2%, increasing to 100% sensitivity and 95.2% specificity when mild steatosis was excluded. However, this varies between studies (with cutoffs in various studies ranging from 1.24–2.2) and is subject to inter-observer variability [71, 117, 118].

Fat quantification techniques using ultrasound attenuation imaging have shown that attenuation imaging is a more accurate method of classifying steatotic livers in adults than B-mode ultrasound [111]. The attenuation coefficient with a high-frequency transducer in children has been shown to positively correlate with MR fat fraction (coefficient 0.498), with a cutoff of 0.699 dB/cm/MHz, resulting in excellent sensitivity and specificity for diagnosing fatty liver (up to 90.2 and 100%, respectively) [119–121].

### Pancreas

As with the liver, fat deposition also occurs in the pancreas of children. In the context of obesity, increased fat intake leads to increased lipolysis, with a correlative increase in free fatty acids, which are then deposited in the pancreatic tissue. This impacts the function of beta cells, leading to reduced insulin secretion and thus metabolic syndrome [122]. Assessment of pancreatic fat by biopsy is invasive and carries risks, so a ratio of the pancreatic body brightness to that of perihepatic fat can be calculated the pancreatico-perihepatic fat index (PPHFI) in determining the presence of metabolic syndrome [123]. The methodology is meticulous, encompassing simultaneous transverse and longitudinal scans to adequately capture pancreatic parenchyma and perihepatic fat. The process includes the careful delineation of elliptical ROIs on the images to measure pancreatic and perihepatic fat brightness. Subsequently, PPHFI is calculated, reflecting the mean brightness ratio of pancreatic to perihepatic fat. This approach is similar to the HRI concept mentioned earlier in the context of liver assessment. In adults, PPHFI has been shown to have a sensitivity of 96% and specificity of 69.7% with a cutoff value of 2.34, with sensitivity decreasing to 77.3% and specificity increasing to 71% when a cutoff of 1.97 was used [124, 125]. Studies in adults have also compared pancreatic echogenicity to that of the kidney and spleen [125–130]. In children, PPHFI has been evaluated in a comparison between 45 participants with obesity and 19 non-obese controls, focusing on the assessment of pancreatic fat. PPHFI showed a higher average of 3.65 in the obese group, significantly exceeding the 0.94 average in the control group, with a strong correlation between ultrasound derived PPHFI and MR-derived pancreatic fat fraction in obese children. The study highlighted that the PPHFI values were significantly higher in the obese group compared to the control, underscoring the potential of ultrasonography as an effective tool for assessing pancreatic fat in pediatric obesity cases [131]. Despite these promising findings, the application of ultrasound for pancreatic fat quantification in pediatric obesity remains an area in development.

### Kidney

Fat quantification in the kidneys uses pararenal and perirenal ultrasonographic fat thickness (PUFT), an important measure in ultrasonography that assesses the fat layers around the kidneys. This method is especially useful in the context of obesity, as it helps determine the amount of visceral fat. Visceral fat is a known risk factor for several metabolic disorders, and its accumulation around the kidneys is particularly concerning [132–134]. PUFT serves as a potential indicator of this fat accumulation, and research has suggested that higher PUFT measurements could independently predict renal impairment in obese individuals [135]. This means that individuals with greater fat thickness around their kidneys, as indicated by PUFT, might be at a higher risk of kidney damage or dysfunction. In comparison to traditional anthropometric measurements like body mass index and waist circumference, which are commonly used to assess obesity, PUFT might provide a more direct and effective correlation with visceral fat [136].

### Bowel

In the context of pediatric ultrasound imaging for bowel assessment, the quantification of fat deposition in the bowel wall is an area of interest [137]. This technique is particularly relevant for conditions such as inflammatory bowel disease, where bowel wall fat deposition—particularly in the submucosa—is seen. The presence of this fat is correlated with poor long-term outcomes, so this represents an important prognostic factor [138]. High-resolution ultrasound allows the capture of detailed images of the bowel and mesenteric fat, with qualitative changes, such as increasing echogenicity, and quantitative changes, such as increased fat thickness, appreciable [139]. These changes can be used to monitor disease progression and response to therapy.

## Summary

This review has given an overview of the emerging modalities in ultrasound and their clinical uses in the pediatric abdomen. Whilst some, such as elastography and qualitative contrast ultrasound, have widespread clinical uptake, other approaches, such as quantitative techniques in MVI and CEUS, and fat quantification, are more novel, and hold great promise for future translational research.

## Data Availability

No datasets were generated or analysed during the current study.
